# Healthy life years among people with and without diabetes in Germany

**DOI:** 10.25646/8331

**Published:** 2021-06-16

**Authors:** Jens Baumert, Christin Heidemann, Lukas Reitzle, Christian Schmidt

**Affiliations:** Robert Koch Institute, Berlin, Department of Epidemiology and Health Monitoring

**Keywords:** DIABETES, HEALTH IMPAIRMENTS, HEALTHY LIFE YEARS, BURDEN OF DISEASE, DIABETES SURVEILLANCE

## Abstract

In addition to life expectancy, the length of time a person can expect to remain free of health-related functional impairments is becoming increasingly important both for the individuals concerned and for society at large. The indicator healthy life years used for this purpose is a key figure for mapping mortality and morbidity. Diabetes is one of the most common chronic diseases and can be associated with health-related functional impairments. In 2014, women and men with diabetes could expect to have significantly fewer healthy life years than people without diabetes; this particularly applies to younger and middle-aged groups. Among 30- to 34-year-olds, for example, women and men with diabetes could expect eleven and twelve fewer healthy life years respectively than people without diabetes. These differences narrow with increasing age. Ensuring that people with and without diabetes have a similar length of lifetime free of health impairments is an important task for public health.

## Introduction

Diabetes mellitus is one of the most common chronic diseases and its prevalence is increasing throughout the world [1]. Diabetes and its often serious comorbidities and secondary diseases [2], which both are associated with increased mortality [3], require lifelong medical treatment and care. In the past few decades, improvements in health care have contributed to lower mortality among people with diabetes [4, 5] and in turn, to an increasing life expectancy [6, 7]. Nevertheless, people with diabetes still have a lower life expectancy than people without diabetes [6, 8].

The positive developments in life expectancy in Germany have also contributed to an increase in the number of years spent with diabetes [7] and in a higher prevalence of health-related functional impairments [9]. These impairments can result from the comorbidities and secondary diseases associated with diabetes which are often serious [10]. Although people with diabetes now live longer than in previous decades, they also have health problems for longer periods of their life. Increasing life expectancy is making the length of time spent without health-related impairments more important at both an individual and societal level. Health impairments primarily involve difficulties in performing everyday activities (e.g. getting dressed, washing or moving, eating and drinking and taking medication). Considerable impairments here contribute to a significantly reduced health-related quality of life [11].

The European Statistical Office (Eurostat) has defined the “Healthy Life Years” (HLY) indicator as a European structural indicator and as a key figure in studying mortality and morbidity [12]. A central goal for public health in dealing with diabetes, therefore, is ensuring that people with diabetes have a similar number of healthy life years than people without diabetes.

Data on healthy life years among younger and middle-aged people with diabetes have not been available for Germany so far. This gap could be closed within the framework of the diabetes surveillance which has been established at the Robert Koch Institute since 2015. The aim of this article is to compare the figures for healthy life years for people with and without diabetes over a broad age spectrum and for both sexes.

## Indicator

The indicator “Healthy Life Years” is defined here as the number of years of life that a person with diabetes can expect to have without health-related functional impairments compared to people without diabetes. For the calculation of this indicator, the prevalences of known diabetes and of health-related functional impairments are taken from the German Health Update (GEDA) 2009, 2010 and 2012 (n=52,112), nationwide telephone health surveys carried out by the Robert Koch Institute. Mortality rates among the general population in 2014, which are also used for the calculation, were provided by the Federal Statistical Office (full survey). The 2014 relative diabetes-related mortality risks are based on health care data from all statutory health insurers (provided in accordance with Germany’s Data Transparency Ordinance, DaTraV, n=47.3 million, population aged 30 years or above). The figures set out in the following are for people aged 30 years or above.

Information about the prevalence of known diabetes was assessed in the GEDA surveys using the question, ‘Have you ever been diagnosed with diabetes by a doctor?’; known diabetes is assumed when respondents answer, ‘Yes’. Data on the prevalence of health impairments is gathered using the question, ‘To what extent do you face permanent restrictions to your daily activities by illness? By permanent we mean for at least six months’; a health impairment is assumed when respondents answer ‘severely limited’. The other possible answers ‘limited but not severely’ and ‘not limited’ form the complementary group. Calculations of the prevalence of diabetes and health impairments were undertaken using summarised data from three GEDA surveys (2009–2012). Weighting factors were used to correct the sample for different selection probabilities and for deviations from the population structure (as of 31 December 2011) with regard to sex, age, education and region. A detailed description of the methodology used in the GEDA surveys 2009, 2010 and 2012 is available in earlier publications [13–15].

The relative diabetes-related mortality risks were calculated using the ratio of the mortality of people with documented diabetes to that of people without documented diabetes. Documented diabetes is defined as a confirmed inpatient diagnosis in at least one quarter or a confirmed documented outpatient diagnosis (E10 to E14) in at least two quarters of one year among people with statutory health insurance. A detailed description of the methodology and preparation of the health care data from statutory health insurers can be found online and in an earlier publication [16, 17].

The estimates of healthy life years were calculated in three steps: first, age-specific mortality rates for people with and without diabetes were calculated using age-specific data on mortality rates among the general population, diabetes prevalence and diabetes-related relative mortality risks. Second, diabetes-specific mortality rates were used to calculate the life expectancies of people with and without diabetes. Finally, the Sullivan method [18] was used to estimate healthy life years using data on age-specific life expectancy and the age-specific prevalence of health impairments.

## Results and discussion

Between 2009 and 2012, the prevalence of diabetes among women and men aged 30 years or above was 10.4%; the prevalence of health impairments was 13.5% among women and 12.3% among men. These prevalences increase significantly with age. The highest prevalence of diabetes is found among 80- to 84-year-olds at 22.0% in women and 24.6% in men. Women and men aged 90 years or above have the highest prevalence of health impairments (33.8% and 32.5%).

**Figure 1:**
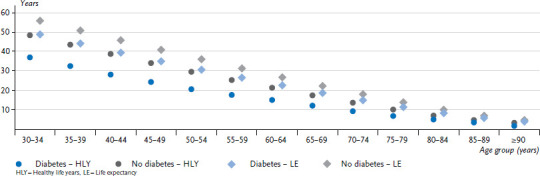
Remaining healthy life years and life expectancy among women aged 30 years or above without and with diabetes by age group in 2014 Source: GEDA 2009, GEDA 2010, GEDA 2012, Causes of Death Statistics from the Federal Statistical Office 2014, DaTraV data 2013/2014

In 2014, women with diabetes (Figure 1) aged 30 to 34 years could expect 36.4 additional healthy life years; men in this age group could expect a further 32.4 healthy life years. Women aged 50 to 54 years could expect 20.3 healthy life years and men 18.7. Women aged 70 to 74 years could expect 9.2 healthy life years and men 8.5. People with diabetes can expect significantly fewer healthy life years than people without diabetes, and this particularly applies to younger and middle-aged groups (Table 1). The difference between the two groups is 11.2 years for women and 11.7 years for men aged 30 to 34 years; 8.8 and 7.4 years among 50- to 54-year-olds, and 4.3 and 3.4 years among people aged 70 to 74 years. Women can expect more healthy life years than men in all age groups, irrespective of their diabetes status. Among people aged 30 to 34 years, a 4.0-year difference in healthy life years was identified between women and men with diabetes and 3.5 for those without diabetes. This difference decreases with age and is lower than twelve months as of 80 years-of-age.

**Figure 2:**
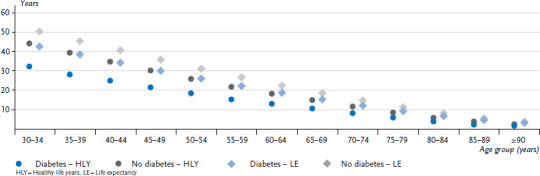
Remaining healthy life years and life expectancy among men aged 30 years or above without and with diabetes by age group in 2014 Source: GEDA 2009, GEDA 2010, GEDA 2012, Causes of Death Statistics from the Federal Statistical Office 2014, DaTraV data 2013/2014

Women aged 30 to 34 years with diabetes have a remaining life expectancy of 48.0 years (Figure 1), whereas the life expectancy among men is 42.6 years (Figure 2). However, women with diabetes in this age group still have around twelve fewer healthy life years, and men with diabetes around ten fewer healthy life years than their peers without diabetes. Healthy life years make up 75.8% of the remaining life expectancy for women with diabetes aged 30 to 34 years and 86.7% of the remaining life expectancy for women without diabetes; the figures are similar for men at 76.0% and 87.8%. These figures decrease equally for both sexes with rising age, irrespective of diabetes status.

Diabetes can be associated with serious comorbidities and secondary diseases [2] that can result in significant health-related functional impairments [8], premature mortality [19] and fewer healthy life years. The present study indicates that women and men with diabetes in Germany can expect significantly fewer healthy life years than those without diabetes, and that this finding particularly applies to younger and middle-aged groups. An earlier study of people aged 65 years or above using relative diabetes-related mortality risks from the 12-year mortality follow-up of the German National Health Interview and Examination Survey 1998 (GNHIES98) in place of DaTraV data but also using prevalences of health impairments from the GEDA surveys 2009 to 2012, as is the case with the present study, reported similar results [19].

When discussing the results presented here in the context of the literature, it should be noted that the instruments used for assessing health impairments and definitions of the construct ‘health impairment’ can differ significantly. This means that a direct comparison of the figures on healthy life years is often only possible to a limited extent. With respect to differences in healthy life years between people with and without diabetes, similar figures to those differences presented here have been identified by other studies [8, 20, 21], even though they used other instruments (e.g. Activities of Daily Living, ADL, and Instrumental Activities of Daily Living, iADL) and observation periods and different prevalences of health-related impairments. Irrespective of their diabetes status, women can expect to have a higher life expectancy as well as to have more life years free of health impairments than men. These differences between the sexes have been observed worldwide for a long time and are mainly explained by biological and behavioural factors [22]. With increasing age, the remaining length of time that people with or without diabetes can expect to remain free from health impairments becomes similar. This is to be expected, due to people’s limited lifespan.

In summary, the figures for healthy life years and life expectancy are significantly lower for women and men with diabetes than those without diabetes, and this finding particularly applies to younger and middle-aged groups. Women can expect more healthy life years and a longer life expectancy than men, regardless as to whether they have diabetes or not. Improvements in diabetes care will be necessary in order to reduce the severity of health-related functional impairments.

Within the framework of the diabetes surveillance, future analyses of healthy life years should also focus on identifying particularly disadvantaged groups and regions so as to determine where effective health policy measures need to be put in place to reduce these differences.

## Key statements

- People who have diabetes can expect to have significantly fewer healthy life years than people without diabetes; this particularly applies to younger and middle-aged people.
- The number of healthy life years that people with and without diabetes can expect to have becomes similar with increasing age.
- Women can expect to have more healthy life years than men; this applies to women with and without diabetes.

## Figures and Tables

**Table 1:** Difference between healthy life years for people without and with diabetes aged 30 years or above Source: GEDA 2009, GEDA 2010, GEDA 2012, Causes of Death Statistics from the Federal Statistical Office 2014, DaTraV data 2013/2014

Age group	Women		Men	
	Difference	(95% CI)	Difference	(95% CI)
30–34 years	11.2	(10.3–12.1)	11.7	(10.6–12.9)
35–39 years	10.8	(9.9–11.7)	11.1	(10.1–12.2)
40–44 years	10.4	(9.6–11.3)	9.7	(8.9–10.5)
45–49 years	9.6	(8.8–10.4)	8.6	(7.9–9.4)
50–54 years	8.8	(8.0–9.5)	7.4	(6.7–8.0)
55–59 years	7.5	(6.8–8.2)	6.5	(5.8–7.1)
60–64 years	6.2	(5.5–6.8)	5.2	(4.6–5.7)
65–69 years	5.2	(4.6–5.8)	4.3	(3.7–4.9)
70–74 years	4.3	(3.7–4.9)	3.4	(2.9–4.0)
75–79 years	3.2	(2.6–3.9)	2.6	(2.0–3.2)
80–84 years	2.0	(1.3–2.6)	1.9	(1.2–2.6)
85–89 years	1.2	(0.5–2.0)	1.6	(0.7–2.6)
≥90 years	1.6	(0.4–2.8)	0.8	(-0.7–2.3)

CI = confidence interval
